# Multimodal imaging including semiquantitative short-wavelength and near-infrared autofluorescence in achromatopsia

**DOI:** 10.1038/s41598-018-23919-w

**Published:** 2018-04-04

**Authors:** Alexandre Matet, Susanne Kohl, Britta Baumann, Aline Antonio, Saddek Mohand-Said, José-Alain Sahel, Isabelle Audo

**Affiliations:** 1 Sorbonne Université, INSERM, CNRS, Institut de la Vision, 17 rue Moreau, F-75012 Paris, France; 2 INSERM-DHOS, CIC1423, DHU ViewMaintain, CHNO des Quinze-Vingts, Paris, 75012 France; 3 Institute for Ophthalmic Research, Centre for Ophthalmology, University of Tuebingen, Tuebingen, Germany; 4 Fondation Ophtalmologique Adolphe de Rothschild, Paris, F-75019 France; 5 Académie des Sciences, Institut de France, Paris, F-75006 France; 6 Department of Ophthalmology, The University of Pittsburgh School of Medicine, Pittsburg, PA United States; 7 University College London, Institute of Ophthalmology, London, EC1V 9EL UK

## Abstract

Multimodal imaging provides insights into phenotype and disease progression in inherited retinal disorders. Congenital achromatopsia (ACHM), a cone dysfunction syndrome, has been long considered a stable condition, but recent evidence suggests structural progression. With gene replacement strategies under development for ACHM, there is a critical need for imaging biomarkers to define progression patterns and follow therapy. Using semiquantitative plots, near-infrared (NIR-AF) and short-wavelength autofluorescence (SW-AF) were explored and correlated with clinical characteristics and retinal structure on optical coherence tomography (OCT). In sixteen ACHM patients with genetic confirmation (*CNGA3*, n = 8; *CNGB3*, n = 7; *PDE6C*, n = 1), semiquantitative plots allowed the detailed analysis of autofluorescence patterns, even in poorly fixating eyes. Twelve eyes showed perifoveal hyperautofluorescent rings on SW-AF, and 7 eyes had central hypoautofluorescent areas on NIR-AF, without association between these alterations (*P* = 0.57). Patients with central NIR-AF hypoautofluorescence were older (*P* = 0.004) and showed more advanced retinal alterations on OCT than those with normal NIR-AF (*P* = 0.051). NIR-AF hypoautofluorescence diameter was correlated to patient age (r = 0.63, *P* = 0.009), size of ellipsoid zone defect on OCT (r = 0.67, *P* = 0.005), but not to the size of SW-AF hyperautofluorescence (*P* = 0.27). These results demonstrate the interest of NIR-AF as imaging biomarker in ACHM, suggesting a relationship with age and disease progression.

## Introduction

Achromatopsia is a congenital retinal disorder belonging to the cone dysfunction syndromes^1,2^. It is characterized by partial or complete color vision deficiency, low visual acuity, photophobia and nystagmus of variable degree. Alterations of visual function are present from birth or early infancy, and are classically described as stationary. Electrophysiology testing shows absent or severely reduced cone function, while rod function is preserved. Structural alterations of outer retinal layers at the fovea have been identified by multimodal imaging^3–5^. Causative mutations have been identified in *CNGA3*^6^ and *CNGB3*^7^ encoding the α- and β-subunits of the cyclic nucleotide-gated channel initiating the depolarization of cone photoreceptors during phototransduction, and less frequently in *GNAT2*^8^, *PDE6C*^9^, and *PDE6H*^10^, also involved in the cone phototransduction cascade. Mutations in the *ATF6* gene, a cellular regulator of the unfolded protein response and endoplasmic reticulum homeostasis, have also been recently identified^11^.

Several clinical trials for *CNGA3* or *CNGB3* gene replacement therapy have been recently initiated or will be carried out in a near future^12,13^. In this perspective, a growing number of studies focus on novel clinical endpoints in achromatopsia, extracted from multimodal imaging of the foveal architecture and reflecting the degree of cone photoreceptor integrity^13–17^. To date, features of near-infrared autofluorescence (NIR-AF) and their relationship with short-wavelength autofluorescence (SW-AF) and SD-OCT findings have not been reported in achromatopsia. NIR-AF is acquired after light stimulation at 787 nm and emanates from melanin, melanolipofuscin and other related compounds localized in the retinal pigment epithelium (RPE) and choroid^18^. The normal fovea exhibits on NIR-AF a central hyperautofluorescent spot of approximately one-disc diameter. Combined to short-wavelength autofluorescence (SW-AF), mostly generated by lipofuscin of the RPE and photophores of photoreceptor outer segments^19^, NIR-AF provides valuable *in vivo* information regarding the metabolic status of the retina and choroid.

In addition, several authors have shown that structural alterations on SD-OCT^3,4,20,21^ and SW-AF^17^ are age-dependent, suggesting that NIR-AF findings may also potentially worsen with age.

In this study, we investigated NIR-AF alterations in achromatopsia using semiquantitative autofluorescence plots, and explored possible correlations between NIR-AF and SW-AF patterns, structural alterations on SD-OCT, and clinical characteristics including patient age.

## Results

### Cohort description and genetic findings

Of 21 subjects with a clinical and molecular diagnosis of congenital achromatopsia, 16 patients for whom complete multimodal imaging was acquired in at least one eye, were retrospectively included (five men, eleven women, mean age: 25.1 ± 11.5 years). Two patients were siblings (Cases #13 and #15) and nine patients presented a family history of consanguinity. Mutations were found in *CNGA3* in eight subjects, in *CNGB3* in seven subjects and in *PDE6C* in one subject. New variants of *CNGA3* were identified in one subject, of *CNGB3* in four subjects (which were recently published elsewhere)^22^, and of *PDE6C* in one subject. Among the five excluded cases, mutations were identified in *CNGA3* in one subject, and in *CNGB3* in four subjects. Detailed genetic findings are reported in Table 1.

**Table 1:** Genetic findings identified in 16 patients with congenital achromatopsia.

#	Age (yrs)	Sex	Ethnicity	History of consanguinity	Affected gene	Mutations Nucleotide (Protein)	Reference
**1**	39	F	Caucasian	−	*CNGA3*	c.1306 C > T (p.Arg436Trp) c.1320delG (p.Trp440Cysfs*25)	Wissinger *et al*.^58^ Wissinger *et al*.^58^
**2**	19	F	Northern African	+	*CNGA3*	c.464delA (p.Lys155Argfs*18) c.464delA (p.Lys155Argfs*18)	New variant
**3**	35	M	Northern African	+	*CNGA3*	c.667 C > T (p.Arg223Trp) c.667 C > T (p.Arg223Trp)	Wissinger *et al*.^58^ Wissinger *et al*.^58^
**4**	10	M	Caucasian	+	*CNGA3*	c.847 C > T (p.Arg283Trp) c.1495 C > T (p.Arg499*)	Kohl *et al*.^6^ Burgueño-Montañés *et al*.^59^
**5**	33	F	Northern African	+	*CNGA3*	c.542 A > G (p.Tyr181Cys) c.542 A > G (p.Tyr181Cys)	Wissinger *et al*.^58^ Wissinger *et al*.^58^
**6**	18	F	Indian	+	*CNGA3*	c.1641C > A (p.Phe547Leu) c.1641C > A (p.Phe547Leu)	Kohl *et al*.^6^ Kohl *et al*.^6^
**7**	40	M	Caucasian	−	*CNGA3*	c.661 C > T (p.Arg221*) c.1279 C > T (p.Arg427Cys)	Johnson *et al*^60^. Wissinger *et al*^58^.
**8**	10	F	Northern African	+	*CNGA3*	c.1669G > A (p.Gly557Arg) c.1669G > A (p.Gly557Arg)	Kohl *et al*^6^. Kohl *et al*^6^.
**9**	12	F	Caucasian	+	*CNGB3*	c.1148delC (p.Thr383Ilefs*13) c.1148delC (p.Thr383Ilefs*13)	Kohl *et al*^7^. Kohl *et al*^7^.
**10**	44	F	Northern African	+	*CNGB3*	c.1006 G > T (p.Glu336*) c.1006 G > T (p.Glu336*)	Kohl *et al*^7^. Kohl *et al*^7^.
**11**	29	F	Caucasian	−	*CNGB3*	c.819_826del (p.Arg274Valfs*13) c.1243 C > T (p.Gln415*)	Kohl *et al*.^7^ Mayer *et al*.^22^
**12**	14	M	Caucasian	−	*CNGB3*	c.130-1 G > T (splice site, p.?) c.130-1 G > T (splice site, p.?)	Mayer *et al*^22^.
**13**	21	F	Caucasian	−	*CNGB3*	c.129 + 2 T > C (splice site, p.?) Deletion of exon 3 (p?)	Mayer *et al*.^22^ Mayer *et al*.^22^
**14**	32	F	Caucasian	−	*CNGB3*	c.3 G > A (p.Met1?) c.1148delC (p.Thr383Ilefs*13)	Mayer *et al*.^22^ Kohl *et al*.^7^
**15**	15	F	Caucasian	−	*CNGB3*	c.129 + 2 T > C (splice site, p.?) Deletion of exon 3 (p.?)	Mayer *et al*.^22^ Mayer *et al*.^22^
**16**	31	M	Northern African	+	*PDE6C*	c.857del (p.Lys286fs*16) c.857del (p.Lys286fs*16)	New variant

Patients #13 and #15 were siblings.

yrs = years.

Photophobia and nystagmus were present in n = 12 (75%) and n = 10 (63%) cases, respectively. Color vision alterations were classified as severe in n = 10 cases (63%) and partial in n = 5 cases (31%), suggestive of complete and incomplete achromatopsia, respectively (one subject was referred for electrophysiology testing only and did not undergo color vision testing). Visual acuity in study eyes was comprised between 20/500 and 20/80. Ten and six patients presented a mild-to-moderate myopia (−0.25 to −6.75) or hyperopia (+0.25 to +5.5), respectively. Hyperopia and myopia were equally frequent among patients presenting *CNGA3* mutations (50% and 50%, respectively), while myopia was more frequent than hyperopia among subjects with *CNGB3* mutations (86% versus 14%). There was a strong correlation between visual acuities from the right and left eyes (*r* = 0.82, *P* = 0.001). Detailed clinical characteristics are reported in Table 2.

**Table 2:** Clinical and imaging findings of 16 patients with congenital achromatopsia.

Patient				Symptoms		Visual function			SD-OCT		Foveal autofluorescence	
#	Age (yrs)	Sex	Affected gene	Photophobia	Nystagmus	Color vision alteration	BCVA (Snellen) OD OS	SE OD/OS, D	Stage (Sundaram *et al*.^14^)	Foveal hypoplasia	SW-AF: hyperauto-fluorescent ring	NIR-AF: hypoauto-fluorescent zone
1	39	F	*CNGA3*	+	−	severe	20/200 20/200	−5.25/−4	ISe absence	−	−	+
2	19	F	*CNGA3*	+	+	severe	20/140 20/200	−5.5/−5.75	continuous ISe	−	+	−
3	35	M	*CNGA3*	+	+	severe	20/500 20/250	+0.5/+2	HRZ	−		+
4	10	M	*CNGA3*	+	+	severe	20/200 20/160	+5.25/+5.5	continuous ISe	−	+	−
5	33	F	*CNGA3*	+	−	partial	20/100 20/100	−6.5/−6.75	ISe absence	+	−	+
6	18	F	*CNGA3*	+	+	severe	20/200 20/200	+1.25/+1.25	HRZ	+	+	+
7	40	M	*CNGA3*	−	+	severe	20/400 20/200	+0.25/+0.25	ISe disruption	+	+	−
8	10	F	*CNGA3*	−	+	partial	20/80 20/100	−2.25 / −3.75	ISe absence	+	+	−
9	12	F	*CNGB3*	+	+	partial	20/200 20/200	+0.25/−0.25	ISe disruption	−	+	−
10	44	F	*CNGB3*	+	−−	partial	20/125 20/160	−5.75/−5.75	HRZ	+	+	+
11	29	F	*CNGB3*	+	−	severe	20/200 20/200	−6.75/−5.25	continuous ISe	+	+	−
12	14	M	*CNGB3*	+	+	severe	20/100 20/125	−0.25/−1.25	ISe disruption	+	−	−
13	21	F	*CNGB3*	+	+	partial	20/200 20/125	−0.5/−0.5	ISe disruption	−	+	+
14	32	F	*CNGB3*	−	+	severe	20/160 20/160	−2.25/−2.25	ISe disruption	+	+	+
15	15	F	*CNGB3*	+	−	severe	20/130 20/130	−1/−1.25	continuous ISe	−	+	−
16	31	M	*PDE6C*	+	−	NA	20/100 20/100	+2/+2.25	HRZ	−	+	+

Patients #13 and #15 were siblings.

yrs = years.

BCVA = best-corrected visual acuity.

SE = spherical equivalent.

D = diopters.

SD-OCT = spectral-domain optical coherence tomography.

SW-AF = short-wavelength autofluorescence.

NIR-AF = near-infrared autofluorescences.

OD = right eye.

OS = left eye.

ISe = inner segment ellipsoid.

HRZ = hyporeflective zone.

### Characteristics of SD-OCT, NIR-AF and SW-AF

**Figure 1:**
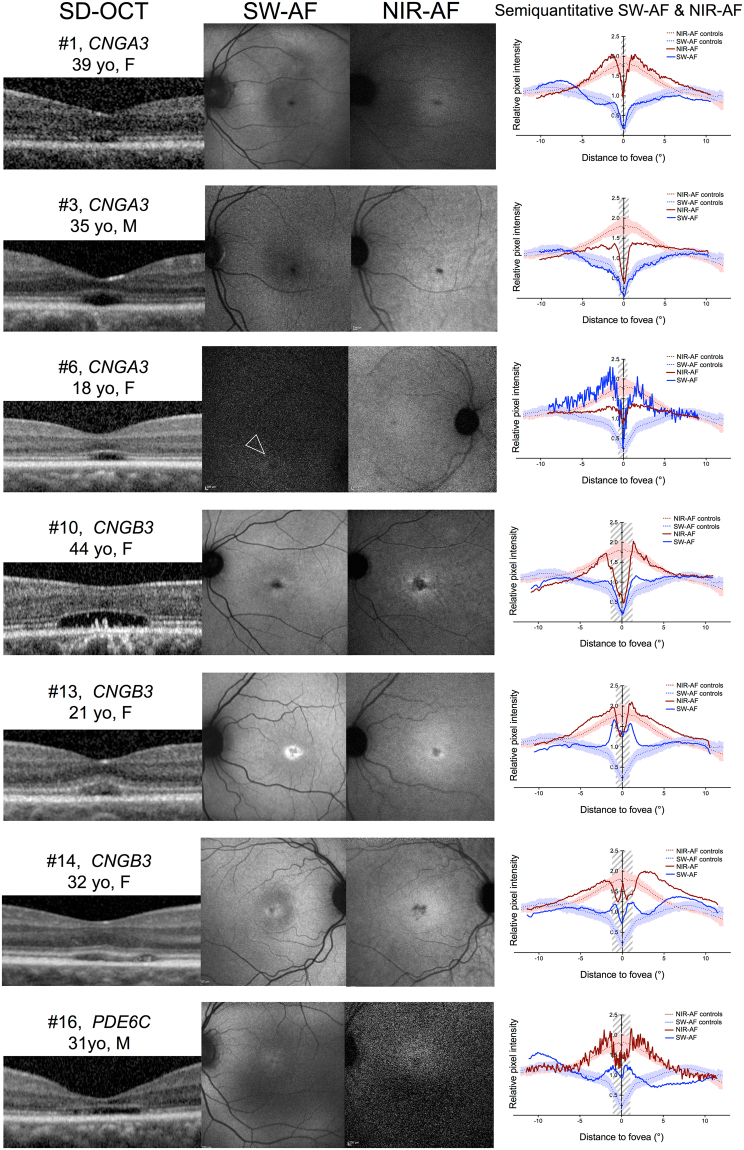
Multimodal imaging in eight patients with achromatopsia showing the spectrum of abnormal near-infrared autofluorescence, and corresponding optical coherence tomography and short-wavelength autofluorescence images. Semiquantitative autofluorescence plots (right) show normalized autofluorescence signals, plotted after segmentation along semi-circles centered on the fovea. The horizontal dimension of the inner segment ellipsoid interruption on optical coherence tomography, if present, is reported as a striped area on autofluorescence plots. In Case #6, a hyperautofluorescent perifoveal ring was barely visible on short-wavelength autofluorescence (white arrowhead) but was clearly visible on the semiquantitative plot, illustrating the interest of this image process to emphasize details in low-quality, noisy images. SD-OCT = spectral domain optical coherence tomography; SW-AF = short-wavelength autofluorescence; NIR-AF = near-infrared autofluorescence; yo = year old; M = male; F = female.

**Figure 2:**
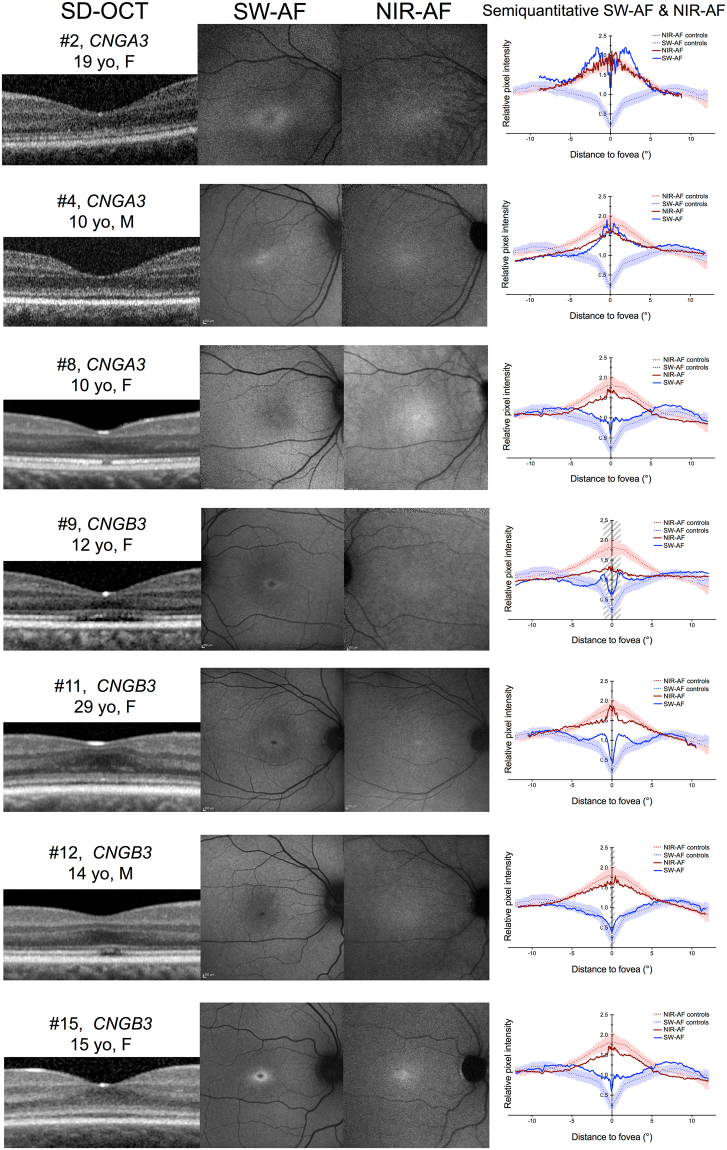
Multimodal imaging in eight patients with achromatopsia displaying normal near-infrared autofluorescence features, and corresponding optical coherence tomography and short-wavelength autofluorescence images. Semiquantitative autofluorescence plots (right) show normalized autofluorescence signals, plotted after segmentation along semi-circles centered on the fovea. The horizontal dimension of the inner segment ellipsoid interruption on optical coherence tomography, if present, is reported as a striped area on autofluorescence plots. SD-OCT = spectral domain optical coherence tomography; SW-AF = short-wavelength autofluorescence; NIR-AF = near-infrared autofluorescence; yo = year old; M = male; F = female.

Multimodal imaging of the 16 cases with achromatopsia are provided in Figs 1, 2, and in the Supplementary Figure 1, and the main imaging features are summarized in Table 2. Complete multimodal imaging acquisition was obtained in the right eye for ten subjects, and in the left eye for six subjects. The eight control subjects selected for the semiquantitative NIR-AF and SW-AF plots did not differ from the 16 achromatopsia cases in terms of age (26.8 versus 25.1 years, *P* = 0.78) and gender (male:female ratio of 3:5 versus 5:11, *P* = 1.0).

According to the classification by Sundaram *et al*.^14^, horizontal SD-OCT scans through the fovea showed a continuous ISe in four patients, an ISe disruption in five patients, an absent ISe in three patients and an hyporeflective zone (HRZ) in four patients. A foveal hypoplasia, defined by the persistence of ≥1 retinal layer more internal than the outer nuclear layer at the fovea, was observed in eight patients. In all patients, the structural alteration on SD-OCT was graded similarly in both eyes.

On SW-AF, the most frequent abnormal feature was a pathognomonic perifoveal hyperautofluorescent ring, that was observed in n = 12 subjects (75%). The semiquantitative autofluorescence plots contributed to identify this ring in a low-quality autofluorescence acquisition due to photophobia, nystagmus and poor fixation (Case #6, Fig. 1). The four cases without perifoveal hyperautofluorescent ring on SW-AF had an abnormal foveal SW-AF profile, consisting in an enlarged (Case #5, Supplementary Figure 1) or a steep central SW-AF depression (Cases #1, Fig. 1; #2 and #12, Fig. 2).

On NIR-AF, the usual increased autofluorescence was present at the center of the macula in all eyes, with a variable signal strength ranging from very faint (Case #9, #11, Fig. 2) to intense (Case #13, #14, Fig. 1; and #8, Fig. 2). Within the macular hyperautofluorescent area, the most frequent abnormal feature of NIR-AF was a central hypoautofluorescent spot of variable size, that was observed in n = 7 subjects (44%), all displayed in Fig. 1. This central hypoautofluorescence, visible on the NIR-AF images, was confirmed by the semiquantitative NIR-AF plots, where it appeared as a central depression whose lowest value reached below the values of the control curve. Among those subjects, two presented in addition a perifoveal NIR-AF hyperautofluorescent ring, colocalizing with the border of the hypoautofluorescent area, as demonstrated by the sharp demarcation visible on semiquantitative autofluorescence plots (Cases #10 and #13, Fig. 1). The remaining cases that did not harbor a central hypoautofluorescent area on NIR-AF are displayed in Fig. 2 (normal appearance of the NIR-AF, n = 7) and in the Supplemental Figure 1 cases, #5 and #7, with borderline NIR-AF plots displaying an ill-defined central decrease in NIR-AF, that could not be clearly categorized into either group). Noticeably, a discrete foveal hyperautofluorescent spot was observed in n = 3 cases (Case #8, #11 and #15, Fig. 2) on the NIR-AF plots.

There was no difference in dimension of the outer retinal defect on SD-OCT between the right and left eyes, when images were available (1.12 ± 0.92 vs 1.08 ± 1.02 degrees, respectively, *P* = 0.70). There was no difference either between the right and left eyes, when imaged, regarding the dimension of the SW-AF hyperautofluorescent perifoveal ring (1.51 ± 1.15 vs 1.32 ± 1.14 degrees, respectively, *P* = 0.56), and the dimension of the NIR-AF central hypoautofluorescence (1.10 ± 1.32 vs 1.11 ± 1.21 degrees, respectively, *P* = 0.84).

There was an excellent intra-observer agreement regarding the measure of the outer retinal defect on SD-OCT images (HRZ or absent ISe), and of the NIR-AF hypoautofluorescence and the SW-AF hyperautofluorescent ring on the semiquantitative plots. The inter-observer agreement was excellent for the SD-OCT evaluation, and substantial for the SW-AF and NIR-AF. Intra- and inter-observer analyses are reported in Table 3.

**Table 3:** Intra- and inter-observer agreement for the assessment of quantitative parameters extracted from multimodal retinal imaging in 16 achromatopsia patients.

	Intra-observer agreement				Inter-observer agreement between observers 1 & 2	
	Observer 1		Observer 2			
	ICC	95% CI	ICC	95% CI	ICC	95% CI
Size of hyper-autofluorescent ring on SW-AF, degree	0.997	0.992–0.999	0.998	0.996–0.999	0.952	0.871–0.983
Size of central hypo-autofluorescence on NIR-AF, degree	0.998	0.994–0.999	0.996	0.990–0.999	0.957	0.885–0.985
Size of ISe alteration on SD-OCT, degree	0.999	0.996–1.0	0.997	0.992–0.999	0.980	0.944–0.993

Agreement was assessed by the intraclass correlation coefficient. The level of agreement was determined by the lower end of the 95% confidence interval, as excellent (>0.90), substantial (0.75–0.90), moderate (0.50–0.75), or poor (<0.50).

ICC = intraclass correlation coefficient.

95% CI = 95% confidence interval.

SD-OCT = spectral-domain optical coherence tomography.

SW-AF = short-wavelength autofluorescence.

NIR-AF = near-infrared autofluorescences.

ISe = inner segment ellipsoid.

### Characteristics of patients with foveal NIR-AF hypoautofluorescence and correlation to multimodal and clinical parameters

Patients presenting a central hypoautofluorescent area on NIR-AF were significantly older than those with normal NIR-AF (31.5 ± 9 versus 15.5 ± 6.6 years, *P* = 0.004). They also presented more frequently advanced outer retinal alterations on SD-OCT, with n = 5/7 cases presenting an absent ISe or an hyporeflective zone as compared to n = 1/7 cases among patients without foveal NIR-AF hypoautofluorescence (*P* = 0.051, near-significant difference). In addition, there was a strong, positive correlation between the size of the NIR-AF central hypoautofluorescent area, measured in degrees on the semiquantitative NIR-AF plots, and the size of the ISe defect, measured in degrees on horizontal SD-OCT scans (*r* = 0.67, *P* = 0.005). This correlation was stronger when restricting the analysis to eyes with central NIR-AF hypoautofluorescence and SD-OCT stage 2 or more (*r* = 0.93, *P* = 0.002). The size of the NIR-AF hypoautofluorescent area was also strongly correlated with the stage of structural damage on SD-OCT (*r* = 0.72, *P* = 0.002). Finally, patient age was also strongly correlated to the size of the hypoautofluorescent area on NIR-AF (*r* = 0.63, *P* = 0.009), and moderately to the stage of SD-OCT alterations (*r* = 0.52, *P* = 0.037), but not to the size of the hyperautofluorescent ring on SW-AF (*P* = 0.89). No relationship was found either between the size of NIR-AF hypoautofluorescence and the SW-AF hyperautofluorescent ring (*P* = 0.27). The diameter of this SW-AF ring was correlated to the size of the ISe defect on SD-OCT (*r* = 0.57, *P* = 0.021), but not the stage of SD-OCT alteration (*P* = 0.48). None of these imaging parameters was correlated to visual acuity levels. Similarly, no correlation was observed between genotype (*CNGA3* or *CNGB3* mutations) and phenotype on multimodal imaging (in term of SW-AF hyperautofluorescent ring, central NIR-AF defect, or SD-OCT grading). The case presenting a homozygous *PDE6C* mutation was not included in this analysis. In particular, although cases with *CNGA3* mutations presented less frequently a perifoveal hyperautofluorescent ring on SW-AF than those with *CNGB3* mutations (n = 5/8 (63%) vs n = 6/7 (86%), respectively), this discrepancy remained non-significant (*P* = 0.57). Multiple correlations are summarized in Table 4.

**Table 4:** Correlation matrix between imaging and clinical parameters in16 patients with congenital achromatopsia.

	P value (Spearman *r*^a^)	Clinical	SD-OCT		SW-AF	NIR-AF
		BCVA, LogMAR	Category (Sundaram *et al*.^14^)	Size of ISe alteration, degree	Size of hyper-autofluorescent ring, degree	Size of central hypo-autofluorescence, degree
Clinical	Age, years	0.60	0.037 (*r* = 0.52)	0.081	0.89	0.009 (*r* = 0.63)
	BCVA, LogMAR		1.0	0.67	0.70	0.84
SD-OCT	Category (Sundaram *et al*.^14^)			0.010 (*r* = 0.63)	0.48	0.002 (*r* = 0.72)
	Size of ISe alteration, degree				0.021 (*r* = 0.57)	0.005 (*r* = 0.67)
SW-AF	Size of hyper-autofluorescent ring, degree					0.27

SD-OCT = spectral-domain optical coherence tomography.

SW-AF = short-wavelength autofluorescence.

NIR-AF = near-infrared autofluorescences.

ISe = inner segment ellipsoid.

^a^Spearman correlation coefficient (reported when *P* ≤ 0.05).

## Discussion

In this study, we report abnormal NIR-AF features in achromatopsia, using semiquantitative autofluorescence plots segmented over semi-circles around the fovea. In addition, we employed this method to correlate NIR-AF with SW-AF features and structural alterations on SD-OCT. Patients with a central hypoautofluorescence on NIR-AF were older and presented more advanced structural lesions at the fovea on SD-OCT than those with normal NIR-AF, while SW-AF was not correlated to other multimodal findings. These observations highlight the interest of NIR-AF as imaging biomarker in achromatopsia, and as potential clinical endpoint in upcoming gene therapy trials. To the best of our knowledge, there is no previous report of NIR-AF findings in cone dysfunction syndromes, and we could find no reference to it in a computerized search on the PubMed database.

The potential interest of NIR-AF for future therapeutic studies is supported by several characteristics. First, NIR-AF alterations were independent from SW-AF findings, but were correlated to structural foveal changes on SD-OCT and to patient age, suggesting that they reflect an age-dependent process, although this finding should be verified in longitudinal cohorts. Second, NIR-AF acquisition produces less dazzling than SW-AF, allowing higher-quality imaging in photophobic patients. More comfortable imaging procedures are also likely to improve patient fixation, and reproducibility of imaging.

Noticeably, this study did not identify correlations between visual acuity and multimodal imaging findings, which may result from the narrow distribution of visual acuity levels among achromatopsia patients, as displayed in Table 2. More complex visual explorations, such as microperimetry testing, which is altered in achromatopsia^5^, may identify relationships with imaging abnormalities but their reliability will be also limited by the unstable fixation in these patients. No significant relationship was identified between genetic and imaging features, in particular regarding SW-AF and NIR-AF patterns, a finding consistent with the absence of genotype/phenotype correlation among achromatopsia subjects^23,24^.

The method providing semiquantitative autofluorescence plots employed here was optimized from previous studies reporting the topographic distribution of autofluorescence, averaged along a horizontal rectangle^17,25,26^. Averaging the autofluorescence signal over circles around the fovea increased the sensitivity to detect subtle autofluorescence changes, as exemplified by Case #6 (Fig. 1), and was particularly suitable to study achromatopsia, where both NIR-AF and SW-AF defects follow circular perifoveal patterns. Moreover, patients with cone dysfunction syndromes often present severe nystagmus and photophobia, limiting image quality due to poor fixation, which was partially overcome by using a graphical representation smoothing image artifacts. This graphical representation of relative autofluorescence levels may be employed as a surrogate for quantitative autofluorescence^27–30^, a promising technique providing an absolute measure of fluorescence, but that requires an internal calibration and that is to date restricted to a few clinical research centers worldwide. Moreover, a multi-observer assessment of the semiquantitative plots demonstrated an excellent and substantial intra- and inter-grader agreement, respectively, which supports the robustness of this method.

While SW-AF is known to derive from lipofuscin accumulation in the RPE^31–33^, and partly from photoreceptor outer segments, with recent evidence that photoreceptor dysfunction may focally increase SW-AF^34^, there is less evidence regarding the exact molecular source of the NIR-AF signal. NIR-AF is thought to derive from melanin and melanolipofuscin granules contained in the RPE and inner choroidal layers^18^, and its topography reflects the higher concentration of these compounds in the macular area. To date, the clinical applications of NIR-AF are limited, although alterations of NIR-AF have been demonstrated in eyes with retinitis pigmentosa^35–37^, Stargardt disease^38–41^, age-related macular degeneration^42–44^, central serous chorioretinopathy^45^ and choroidal nevi^46–48^, the latter confirming that melanin is the predominant source of the NIR-AF signal. The association of NIR-AF with quantitative^27,49,50^ or lifetime autofluorescence techniques^51^, so far developed for SW-AF, may enhance the reproducibility and comparability of NIR-AF.

Historically, there has been only two pathology reports of eyes with presumed achromatopsia^52,53^, both showing cone photoreceptor loss, ectopic cone nuclei, and an abnormal morphology of photoreceptor outer segments, consistent with modern SD-OCT imaging. Moreover, outer segments were positively labelled by periodic acid-Schiff staining, highlighting the presence of glycoproteins and glycolipids. We hypothesize that this finding manifests the accumulation of degradation products within cone outer segments due to the defective phototransduction cascade. This would provide an explanation for both the abnormal SW-AF signal, via accumulation of degradation compounds in the RPE, and NIR-AF alterations, via secondary changes in the polarized distribution of RPE melanin/melanolipofuscin granules^54^ induced by the uptake of these excessive byproducts. In addition, melanin has a photoprotective role in RPE cells^54^, and its altered distribution may further aggravate retinal phototoxicity and central RPE lesions, and could also contribute to the photophobia experienced by achromat subjects. Interestingly, previous reports have also identified NIR-AF alterations in several macular disorders where photoreceptor function is altered, like retinitis pigmentosa^35–37^, or Stargardt disease^38–41^, resulting in RPE modifications visualized on NIR-AF. Moreover, melanin within the choroid also contributes to the NIR-AF signal^18^. Severe, permanent alterations in cone metabolism is likely to induce changes in choroidal supply and uptake of oxygen and nutrients, at the level of the macula.

Several retrospective studies have identified a possible age-dependency of structural alterations in achromatopsia. Thiadens *et al*. reported that ISe interruption was more frequent in older patients, and that central retinal thickness correlated with age^3^. Thomas *et al*. observed a relationship between HRZ and outer nuclear layer thinning with age^4^. In a cohort of nine affected children aged 8 years or less, Yang *et al*. showed that the foveal morphology is relatively preserved in early childhood, with limited thinning of retinal layers compared to age-matched controls^20^. However, these results were based on cross-sectional observations, with limited longitudinal evidence. Lee *et al*. compared ten children with achromatopsia, seven of which were followed longitudinally, with 59 matched controls, and observed a reduced growth of outer retinal layers compared to inner layers, indicating that the normal retinal development is impaired in achromatopsia eyes^21^. This finding is consistent with the high rate of foveal hypoplasia in these patients, observed in the present cohort (Table 2) and previously reported^55^. In a prospective study, Aboshiha *et al*^16^. have reported a time-dependent progression of structural alterations in two of 37 patients (5%) using serial SD-OCT, and a discrete enlargement of the central SW-AF lesion in a subgroup with marked central hypoautofluorescence, but these results were based on a relatively short mean follow-up of 19.5 months, and require additional confirmation. Regarding fundus autofluorescence findings, Fahim *et al*. have reported qualitatively that a sharply demarcated central hypofluorescent lesion on SW-AF seems to be more frequent in older patients^17^. Longitudinal reports on disease progression assessed by SD-OCT or confocal fundus autofluorescence are limited by the relatively recent availability of these high-resolution multimodal imaging tools. Interestingly, cone dysfunction syndromes, including achromatopsia, were classically described as stationary disorders^1^, which has become subject of debate given the increasing evidence of structural progression presented above^16^. Yet, functional progression over time has been seldom reported. For instance, Thiadens *et al*. observed in a retrospective longitudinal cohort that visual acuity deteriorated below 20/200 between childhood and adulthood in 10 of 81 cases (12%)^23^.

This study presents several limitations, including its retrospective, cross-sectional nature and the small cohort size, inherent to rare inherited retinal disorders. However, this small number of cases underwent standardized, comprehensive multimodal imaging in a tertiary retinal dystrophy clinic, and their molecular diagnosis was ascertained in a reference genotyping center for achromatopsia, after exclusion of those with incomplete imaging or unclear diagnosis. Image quality was limited by low visual acuity, photophobia, nystagmus, poor fixation, and young age, which are also inherent characteristics of achromatopsia patients. These limitations were compensated for by exclusion criteria based on the patients’ ability to undergo the image acquisition procedure, and by the segmentation method applied to SW-AF and NIR-AF images. The main limitation of this semi-circular segmentation of the autofluorescence signal is the splitting of the superior and inferior hemi-regions which are averaged into the temporal (from 12 to 6 o’clock, temporally) and nasal (from 12 to 6 o’clock, nasally) hemi-circular regions, artificially reducing their weight in the graphical representation. Yet, the technique improves previously reported methods^17,25,26^ that only accounted for the nasal and temporal regions within a horizontal rectangular region of interest. Moreover, quantitative measurements can be subjective, particularly due to limited image quality in some cases, and noise on the semiquantitative plots, potentially affecting statistical analyses. Therefore, 2-observer measurements were obtained, with excellent intra- and inter-observer agreement, supporting the analysis. Finally, longitudinal follow-up data was only available for a small proportion of subjects, with different inter-visit intervals, which did not warrant a methodologically relevant interpretation for this limited longitudinal data. Future prospective studies with longitudinal assessment, and focusing on the reliability and repeatability of quantitative SW-AF and NIR-AF measurements, are therefore needed to assess the time-dependent structural and imaging changes suggested in the present work.

To summarize, the present results suggest that semiquantitative plots contribute to visualize autofluorescence imaging patterns, especially in eyes with photophobia or limited fixation, as those affected by cone dysfunction syndromes. Moreover, NIR-AF imaging is a promising biomarker for the imaging of achromatopsia, and a potential surrogate endpoint^56^ for future clinical trials.

## Methods

### Subjects

This retrospective observational study involving human subjects adhered to the tenets of the Declaration of Helsinki, and was performed in accordance with local regulations. Written informed consent was obtained from all subjects after a full explanation of the clinical and genetic procedures was provided. Ethics Committee approval was obtained (Ethics Committee of the Paris Region: “Comité de Protection des Personnes Ile-de-France V”). The study included consecutive patients evaluated between 2009 and 2014 at the retinal dystrophy clinic, Quinze-Vingts Hospital (Paris, France), with a clinical diagnosis of achromatopsia confirmed by genetic testing, and for whom complete multimodal imaging with NIR-AF, SW-AF, and SD-OCT had been acquired in at least one eye. Subjects were excluded when acquisition of these three imaging modalities was impossible in both eyes, due to poor fixation and/or photophobia. The clinical diagnosis relied on a history of altered color perception, confirmed by color vision testing, and low visual acuity since early childhood, and absent photopic responses with normal scotopic responses on electroretinography, suggesting a cone dysfunction syndrome. Color vision was tested by the 15-Hue Farnsworth test and was classified as “partial” in case of ≤3 errors or “severe” in case of ≥4 errors. Electroretinography was performed using the Espion system (Diagnosys LLC, Lowell, MA) according to the International Society for Clinical Electrophysiology of Vision (ISCEV) protocol^57^.

Eight healthy individuals of comparable age underwent SW-AF and NIR-AF to serve as controls for the graphical representation of autofluorescence plots.

### Genetic testing

DNA samples were stored at the NeuroSensCol DNA bank for research in neuroscience at the CHNO des Quinze-Vingts, Inserm, CNRS, Paris, France (Principal investigator (PI): JAS, co-PI: IA). Genetic testing was performed at the Molecular Genetics Laboratory, University Eye Hospital, Tuebingen, Germany according to the following strategy: first, the frequent c.1148delC mutation was screened in *CNGB3* in all cases, then L and M cone opsin gene mutations corresponding to blue cone monochromacy were excluded in sporadic males with compatible X-linked recessive heredity, then by order of frequency, direct sequencing of *CNGA3* exons 5–7, *CNGB3* exons 1–18, *CNGA3* exons 1–4, *GNAT2*, *PDE6H*, and then *PDE6C* was performed.

### Multimodal imaging acquisition and processing

SD-OCT, and confocal NIR-AF and SW-AF pictures were acquired with the Spectralis and HRA2 devices (Heidelberg Engineering, Heidelberg, Germany), respectively. Multimodal imaging was acquired in a standardized manner by trained orthoptists at the retinal imaging facility of the Center for Clinical Investigation at our institution, following standard procedures for phenotypic studies of inherited retinal dystrophies. Based on previous reports by Sundaram *et al*. and Aboshiha *et al*. ascertaining the symmetrical presentation of achromatopsia, and allowing the investigation of imaging features in one single eye^14,16^, the right eye of each subject was retained for analysis, except when complete multimodal imaging was only available for the left eye.

NIR-AF and SW-AF images were analyzed with a custom processing based on a modification of the method by Lois *et al*^25,26^. Semiquantitative autofluorescence plots were obtained using the publicly available Image J software (ImageJ 64 version 1.48q, Wayne Rasband, National Institutes of Health, Bethesda, MD, http://imagej.nih.gov/ij) and the “Radial profile angle” plugin (Philippe Carl, http://rsb.info.nih.gov/ij/plugins/radial-profile-ext.html), as illustrated in the Supplementary Figure 2. After conversion to 8-bit grayscale pictures, this plugin calculates mean gray intensity values along concentric circular lines. After visual identification of the fovea, two hemi-circular regions of interest of radius R (in pixels), centered on the fovea were defined, one on the temporal and one on the nasal side of the macula. R was determined as the maximum distance between the fovea and the optic disc or the temporal border of the picture (usually 160–200 pixels). Then the software extracted the mean grayscale pixel intensity for each nasal and temporal hemi-circle of radius 1 to R. After subtraction of the autofluorescence intensity reference *AF*_*ref*_, set manually on the optic nerve head, each grayscale intensity level *AF*_*measured*_ for a radius r (from 1 to R) was divided by the mean grayscale intensity of the full autofluorescence image *AF*_*mean*_ after subtraction of *AF*_*ref*_, in order to provide a normalized relative autofluorescence level *AF* (r), according to the following formula:

$$AF(r)=(A{F}_{measured}(r)-A{F}_{ref})/(A{F}_{mean}-A{F}_{ref})$$

Finally, after pixel-to-degree conversion, relative autofluorescence intensities were exported to GraphPad Prism (version 5.0 f, GraphPad Software, La Jolla, CA, USA), and a combined graphical representation of averaged SW-AF and NIR-AF patterns of a single eye along hemicircular lines was plotted. The standard deviation interval of relative autofluorescence values acquired in the right eye of eight age-matched healthy subjects was also represented along with autofluorescence plots of each investigated eye.

Structural foveal alterations on horizontal SD-OCT scans through the fovea were categorized according to Sundaram *et al*.^14^, as stage 1, continuous inner segment ellipsoid band (ISe); stage 2, ISe disruption; stage 3, ISe absence; and stage 4, hyporeflective zone (HRZ) affecting outer retinal layers.

The average diameter of central fluorescence abnormalities on SW-AF and NIR-AF was measured in degrees using the semiquantitative autofluorescence plots by two independent observers (IA and AM) in a masked fashion. The extension of ISe defects on horizontal SD-OCT scans was measured independently on anonymized images by the two observers using the built-in caliper function of the Heidelberg Eye Explorer software (version 1.9.10.0). Each imaging parameter was measured twice by each observer, to assess intra-observer agreement, and the mean between both measures was used to assess inter-observer agreement. For statistical analyses, the mean between measures by the two observers was employed.

### Statistical analyses

Mann-Whitney test, Fisher’s exact test and Spearman coefficients were used for comparative, contingency and correlations statistics, respectively, on GraphPad Prism (version 5.0 f, GraphPad Software, La Jolla, CA, USA). The Wilcoxon paired test was used to assess inter-eye symmetry. Intra-class correlation coefficients were employed to assess intra- and inter-observer agreement for subjective quantitative measurements. Based on the lower end of the 95%-confidence interval (95CI) of the ICC, agreement was considered as poor (<0.50), moderate (0.50–0.75), good (0.75–0.90), or excellent (>0.90). The “irr” package was used for ICC calculations on R software (Version 3.3.0, R Foundation for Statistical Computing, R Core Team, 2016, Vienna, Austria. http://www.R-project.org/). The logarithm of the minimal angle of resolution (LogMAR) was used for calculations on best-corrected visual acuity levels. *P* values ≤ 0.05 were considered significant.

### Data availability

The clinical data generated and analyzed during the current study are provided in Tables 1 and 2. Retinal imaging data and corresponding semiquantitative plots are provided in Figs 1, 2 and in the Supplementary Figure 1. The datasets employed for statistical analyses are available from the corresponding author on reasonable request. Comprehensive genetic data is provided in Table 1 and available in the public repository ClinVar of the National Center for Biotechnology Information (https://www.ncbi.nlm.nih.gov/clinvar/).

## Electronic supplementary material

Supplementary Information

## References

[1] Michaelides M, Hunt DM, Moore AT (2004). The cone dysfunction syndromes. Br. J. Ophthalmol..

[2] Aboshiha J, Dubis AM, Carroll J, Hardcastle AJ, Michaelides M (2016). The cone dysfunction syndromes. Br. J. Ophthalmol..

[3] Thiadens AAHJ (2010). Progressive loss of cones in achromatopsia: an imaging study using spectral-domain optical coherence tomography. Invest. Ophthalmol. Vis. Sci..

[4] Thomas MG, Kumar A, Kohl S, Proudlock FA, Gottlob I (2011). High-resolution *in vivo* imaging in achromatopsia. Ophthalmology.

[5] Genead MA (2011). Photoreceptor structure and function in patients with congenital achromatopsia. Invest. Ophthalmol. Vis. Sci..

[6] Kohl S (1998). Total colourblindness is caused by mutations in the gene encoding the alpha-subunit of the cone photoreceptor cGMP-gated cation channel. Nat. Genet..

[7] Kohl S (2000). Mutations in the CNGB3 gene encoding the β-subunit of the cone photoreceptor cGMP-gated channel are responsible for achromatopsia (ACHM3) linked to chromosome 8q21. Hum. Mol. Genet..

[8] Kohl S (2002). Mutations in the cone photoreceptor G-protein alpha-subunit gene GNAT2 in patients with achromatopsia. Am. J. Hum. Genet..

[9] Thiadens AAHJ (2009). Homozygosity mapping reveals PDE6C mutations in patients with early-onset cone photoreceptor disorders. Am. J. Hum. Genet..

[10] Kohl S (2012). A nonsense mutation in PDE6H causes autosomal-recessive incomplete achromatopsia. Am. J. Hum. Genet..

[11] Kohl S (2015). Mutations in the unfolded protein response regulator ATF6 cause the cone dysfunction disorder achromatopsia. Nat. Genet..

[12] Zobor D, Zobor G, Kohl S (2015). Achromatopsia: on the doorstep of a possible therapy. Ophthalmic Res..

[13] Zobor D (2017). The Clinical Phenotype of *CNGA3* -Related Achromatopsia: Pretreatment Characterization in Preparation of a Gene Replacement TherapyTrial. Investig. Opthalmology Vis. Sci..

[14] Sundaram, V. *et al*. Retinal Structure and Function in Achromatopsia: Implications for Gene Therapy. *Ophthalmology*10.1016/j.ophtha.2013.08.017 (2013). 10.1016/j.ophtha.2013.08.017 PMC3895408 24148654

[15] Aboshiha J (2014). Dark-adaptation functions in molecularly confirmed achromatopsia and the implications for assessment in retinal therapy trials. Investig. Ophthalmol. Vis. Sci..

[16] Aboshiha J (2014). A prospective longitudinal study of retinal structure and function in achromatopsia. Invest. Ophthalmol. Vis. Sci..

[17] Fahim AT (2013). Diagnostic fundus autofluorescence patterns in achromatopsia. Am. J. Ophthalmol..

[18] Keilhauer CN, Delori FC (2006). Near-Infrared Autofluorescence Imaging of the Fundus: Visualization of Ocular Melanin. Investig. Opthalmology Vis. Sci..

[19] Delori FC (1995). *In vivo* fluorescence of the ocular fundus exhibits retinal pigment epithelium lipofuscin characteristics. Invest. Ophthalmol. Vis. Sci..

[20] Yang, P. *et al*. Retinal Morphology of Patients With Achromatopsia During Early Childhood: Implications for Gene Therapy. *JAMA Ophthalmol*. 10.1001/jamaophthalmol.2014.685 (2014). 10.1001/jamaophthalmol.2014.685 PMC8174570 24676353

[21] Lee FRCOphth H (2015). Retinal Development in Infants and Young Children with Achromatopsia. Ophthalmology.

[22] Mayer, A. K. *et al*. *CNGB3* mutation spectrum including copy number variations in 552 achromatopsia patients. *Hum*. *Mutat*. 10.1002/humu.23311 (2017). 10.1002/humu.23311 28795510

[23] Thiadens AAHJ (2009). Genetic etiology and clinical consequences of complete and incomplete achromatopsia. Ophthalmology.

[24] Aboshiha J, Dubis AM, Carroll J, Hardcastle AJ, Michaelides M (2016). The cone dysfunction syndromes. Br J Ophthalmol.

[25] Lois N, Halfyard aS, Bird aC, Fitzke FW (2000). Quantitative evaluation of fundus autofluorescence imaged ‘*in vivo*’ in eyes with retinal disease. Br. J. Ophthalmol..

[26] Lois N, Halfyard AS, Bird AC, Holder GE, Fitzke FW (2004). Fundus autofluorescence in Stargardt macular dystrophy-fundus flavimaculatus. Am. J. Ophthalmol..

[27] Duncker, T. *et al*. Quantitative Fundus Autofluorescence Distinguishes ABCA4-Associated and Non-ABCA4-Associated Bull’s-Eye Maculopathy. *Ophthalmology*10.1016/j.ophtha.2014.08.017 (2014). 10.1016/j.ophtha.2014.08.017 PMC4306619 25283059

[28] Ach, T. *et al*. Quantitative autofluorescence and cell density maps of the human retinal pigment epithelium. *Invest*. *Ophthalmol*. *Vis*. *Sci*. 10.1167/iovs.14-14802 (2014). 10.1167/iovs.14-14802 PMC4123894 25034602

[29] Müller PL (2015). Monoallelic *ABCA4* Mutations Appear Insufficient to Cause Retinopathy: A Quantitative Autofluorescence Study. Investig. Opthalmology Vis. Sci..

[30] Marsiglia M (2015). Quantitative Autofluorescence as a Clinical Tool for Expedited Differential Diagnosis of Retinal Degeneration. JAMA Ophthalmol..

[31] Sparrow JR (2012). The bisretinoids of retinal pigment epithelium. Prog. Retin. Eye Res..

[32] Greenberg JP (2013). Quantitative fundus autofluorescence in healthy eyes. Invest. Ophthalmol. Vis. Sci..

[33] Sparrow JR (2010). Fundus autofluorescence and the bisretinoids of retina. Photochem. Photobiol. Sci..

[34] Zhao J, Kim HJ, Sparrow JR (2017). Multimodal Fundus Imaging of Sodium Iodate-Treated Mice Informs RPE Susceptibility and Origins of Increased Fundus Autofluorescence. Investig. Opthalmology Vis. Sci..

[35] Cideciyan AV, Swider M, Jacobson SG (2015). Autofluorescence imaging with near-infrared excitation:normalization by reflectance to reduce signal from choroidal fluorophores. Invest. Ophthalmol. Vis. Sci..

[36] Kellner U (2009). Lipofuscin- and melanin-related fundus autofluorescence visualize different retinal pigment epithelial alterations in patients with retinitis pigmentosa. Eye (Lond)..

[37] Duncker T (2013). Comparison of near-infrared and short-wavelength autofluorescence in retinitis pigmentosa. Invest. Ophthalmol. Vis. Sci..

[38] Sparrow JR (2015). Flecks in Recessive Stargardt Disease: Short-Wavelength Autofluorescence, Near-Infrared Autofluorescence, and Optical Coherence Tomography. Invest. Ophthalmol. Vis. Sci..

[39] Duncker T (2014). Correlations among near-infrared and short-wavelength autofluorescence and spectral-domain optical coherence tomography in recessive stargardt disease. Investig. Ophthalmol. Vis. Sci..

[40] Greenstein VC (2015). Near-infrared autofluorescence: its relationship to short-wavelength autofluorescence and optical coherence tomography in recessive stargardt disease. Invest. Ophthalmol. Vis. Sci..

[41] Kellner S (2009). Lipofuscin- and melanin-related fundus autofluorescence in patients with ABCA4-associated retinal dystrophies. Am. J. Ophthalmol..

[42] Kellner U, Kellner S, Weinitz S (2010). Fundus autofluorescence (488 NM) and near-infrared autofluorescence (787 NM) visualize different retinal pigment epithelium alterations in patients with age-related macular degeneration. Retina.

[43] Heiferman MJ, Fawzi AA (2016). Discordance between blue-light autofluorescence and near-infrared autofluorescence in age-related macular degeneration. Retina.

[44] Schmitz-Valckenberg S (2011). Localisation and significance of *in vivo* near-infrared autofluorescent signal in retinal imaging. Br. J. Ophthalmol..

[45] Kim SK, Kim SW, Oh J, Huh K (2013). Near-infrared and short-wavelength autofluorescence in resolved central serous chorioretinopathy: Association with outer retinal layer abnormalities. Am. J. Ophthalmol..

[46] Weinberger AWa (2006). Fundus near infrared fluorescence correlates with fundus near infrared reflectance. Invest. Ophthalmol. Vis. Sci..

[47] Skondra D, Papakostas TD, Hunter R, Vavvas DG (2012). Near infrared autofluorescence imaging of retinal diseases. Semin. Ophthalmol..

[48] Vallabh NA (2016). Near-infrared reflectance and autofluorescence imaging characteristics of choroidal nevi. Eye.

[49] Delori F (2011). Quantitative Measurements of Autofluorescence with the Scanning Laser Ophthalmoscope. Invest. Ophthalmol. Vis. Sci..

[50] Gliem M (2016). Quantitative Fundus Autofluorescence in Early and Intermediate Age-Related Macular Degeneration. JAMA Ophthalmol..

[51] Dysli C (2017). Fluorescence lifetime imaging ophthalmoscopy. Prog. Retin. Eye Res..

[52] Harrison R, Hoefnagel D, Hayward JN (1960). Congenital total color blindness: a clincopathological report. Arch. Ophthalmol..

[53] Falls HF, Wolter JR, Alpern M (1965). Typical total monochromacy. A histological and psychophysical study. Arch. Ophthalmol..

[54] Sarna T (1992). Properties and function of the ocular melanin–a photobiophysical view. J. Photochem. Photobiol. B..

[55] Thomas MG (2011). Structural grading of foveal hypoplasia using spectral-domain optical coherence tomography a predictor of visual acuity?. Ophthalmology.

[56] Villani E, Vujosevic S, RM C (2017). Foreword: Biomarkers and Surrogate Endpoints in OphthalmicClinical Research. Investig. Opthalmology Vis. Sci..

[57] McCulloch DL (2015). ISCEV Standard for full-field clinical electroretinography (2015 update). Doc. Ophthalmol..

[58] Wissinger B (2001). CNGA3 mutations in hereditary cone photoreceptor disorders. Am. J. Hum. Genet..

[59] Burgueño-Montañés, C., Colunga Cueva, M. & Costales Álvarez, C. A novel mutation in the CNGA3 gene responsible for incomplete achromatopsia. *Arch*. *Soc*. *Esp*. *Oftalmol*. 10.1016/j.oftal.2012.07.019 (2012). 10.1016/j.oftal.2012.07.019 24269407

[60] Johnson S (2004). Achromatopsia caused by novel mutations in both CNGA3 and CNGB3. J. Med. Genet..

