# Supplementary material for: Multimodal imaging including semiquantitative short-wavelength and near-infrared autofluorescence in achromatopsia
Source: Sci Rep. 2018 Apr 4;8:5665. doi: 10.1038/s41598-018-23919-w (PMC5884771; doi:10.1038/s41598-018-23919-w)
Supplement: Supplementary file 1 — Supplementary Information [file 41598_2018_23919_MOESM1_ESM.pdf]

Supplementary Information

Manuscript title:

Multimodal imaging including semiquantitative short-wavelength and near-infrared autofluorescence in achromatopsia

Authors:

Alexandre MATET, MD, PhD,[1,2] Susanne KOHL, MD, PhD,[3] Britta BAUMANN,[3] Aline ANTONIO[1], Saddek MOHAND-SAID, MD,[1,2] José-Alain SAHEL, MD,[1,2,5-7] Isabelle AUDO, MD, PhD[1,2,7]*

1. Sorbonne Université, INSERM, CNRS, Institut de la Vision, 17 rue Moreau, F-75012 Paris, France.

2. INSERM-DHOS, CIC1423, DHU ViewMaintain, CHNO des Quinze-Vingts, Paris 75012, France

3. Institute for Ophthalmic Research, Centre for Ophthalmology, University of Tuebingen, Tuebingen, Germany

4. Fondation Ophtalmologique Adolphe de Rothschild, Paris F-75019, France

5. Académie des Sciences, Institut de France, Paris F-75006, France

6. Department of Ophthalmology, The University of Pittsburgh School of Medicine, Pittsburg, PA, United States

7. University College London, Institute of Ophthalmology, London EC1V 9EL, UK

Supplementary Figure 1.

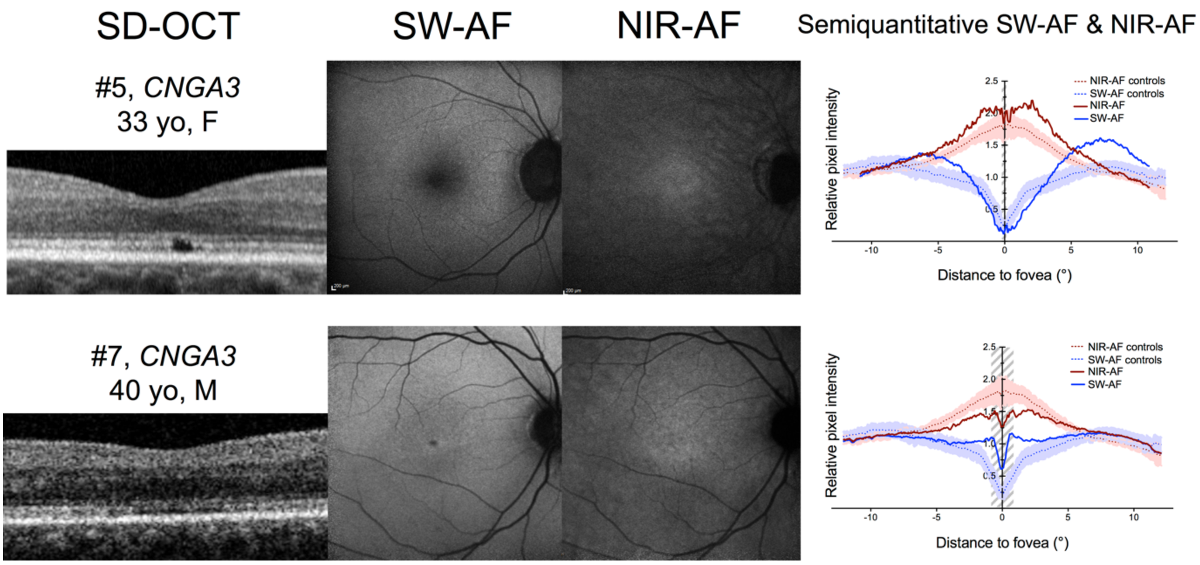

Multimodal imaging in two patients with achromatopsia displaying (from left to right) optical coherence tomography, short-wavelength autofluorescence and near-infrared autofluorescence images. Semiquantitative autofluorescence plots (right) show normalized autofluorescence signals, plotted after segmentation along semi-circles centered on the fovea. The horizontal dimension of the inner segment ellipsoid interruption on optical coherence tomography, if present, is reported as a striped area on autofluorescence plots. The two cases displayed harbor a borderline pattern with ill-defined central near-infrared autofluorescence that did not allow their categorization.

SD-OCT= spectral domain optical coherence tomography; SW-AF= short-wavelength autofluorescence; NIR-AF= near-infrared autofluorescence; yo= year old; M= male; F= female

Supplementary Figure 2.

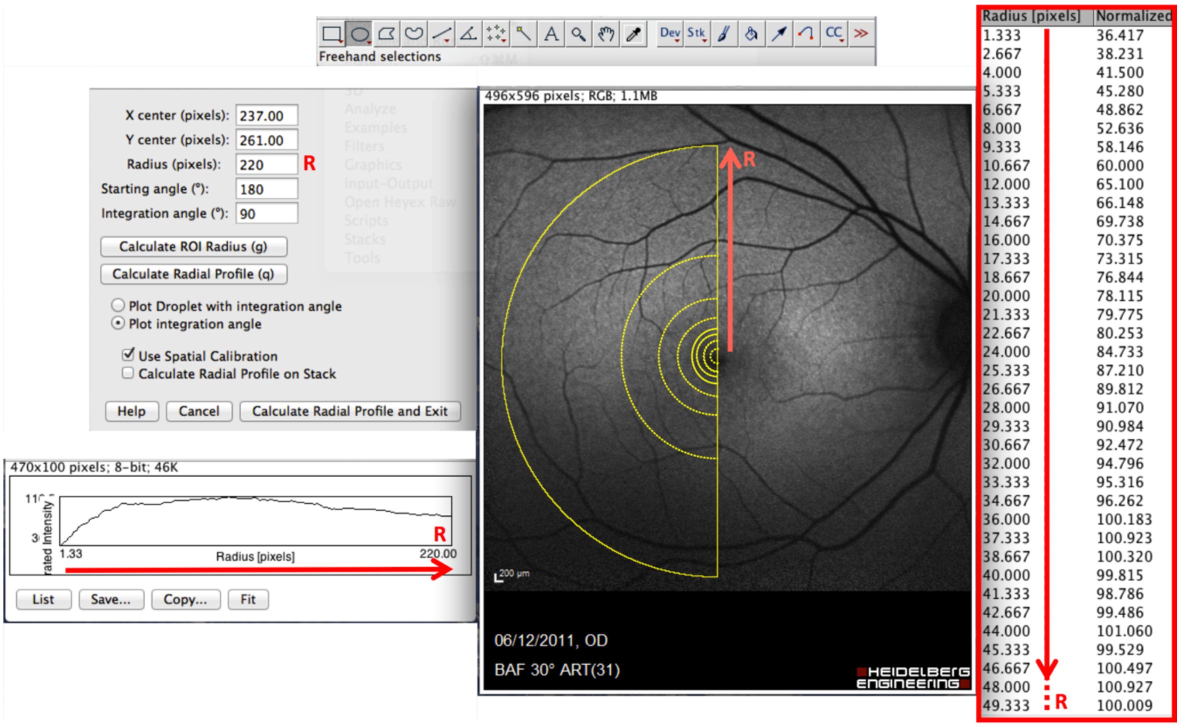

Illustration of the method to obtain semiquantitative autofluorescence plots using the ImageJ software. A temporal semi-circle of radius R (here 220 pixels) was centered on the fovea using the “Radial profile angle” plugin. Grayscale pixel values were averaged along concentric semi-circles from radii r (from 1.3 to 220 pixels). Averaged pixel intensities can be visualized in the lower left window and are provided in the right window (red contour). The process was repeated with a similar nasal semi-circle, and values were exported to a conventional spreadsheet software for graphical representation.
